# Extracellular heat shock protein 70 inhibits tumour necrosis factor-α induced proinflammatory mediator production in fibroblast-like synoviocytes

**DOI:** 10.1186/ar2399

**Published:** 2008-04-14

**Authors:** Xinjing Luo, Xiaoxia Zuo, Yaou Zhou, Bing Zhang, Yongzhong Shi, Meidong Liu, Kangkai Wang, D Randy McMillian, Xianzhong Xiao

**Affiliations:** 1 Department of Pathophysiology, Xiangya School of Medicine, Central South University, Xiangya Road, Changsha, Hunan 410008, China; 2 Department of Laboratory Medicine, Medical College of Taizhou University, Shifu Road, Taizhou, Zhejiang 318000, China; 3 Department of Rheumatology and Clinical Immunology, Xiangya Hospital, Central South University, Xiangya Road, Changsha, Hunan 410008, China; 4 Department of Pediatrics, University of Texas Southwestern Medical Center, 5323 Harry Hines Blvd, Dallas, Texas 75390-9063, USA

## Abstract

**Introduction:**

It was recently suggested that heat shock protein (HSP)70, an intracellular protein, is a potential mediator of inflammatory disease when it is released into the extracellular compartment. Although elevated HSP70 levels have been identified in rheumatoid arthritis (RA) synovial tissues and RA synovial fluid compared with patients with osteoarthritis and healthy individuals, it remains unclear what role extracellular HSP70 plays in the pathogenesis of RA. This study was conducted to investigate the effects of extracellular HSP70 on the production of RA-associated cytokines in fibroblast-like synoviocytes from patients with RA and to elucidate the mechanisms involved.

**Methods:**

IL-6, IL-8 and monocyte chemoattractant protein (MCP)-1 levels in culture supernatants were measured using enzyme-linked immunosorbent assays. Activation of mitogen-activated protein kinases (MAPKs), such as extracellular signal-regulated protein kinases (ERKs), c-Jun amino-terminal kinase (JNK) and p38 MAPK, was detected using Western blotting. Nuclear translocation of nuclear factor-κB (NF-κB) and degradation of the inhibitory protein IκBα were examined using immunohistochemistry and Western blotting.

**Results:**

Human HSP70 downregulated IL-6, IL-8 and MCP-1 production in RA fibroblast-like synoviocytes induced by tumour necrosis factor (TNF)-α in a concentration dependent manner. HSP70 inhibited the activation of ERK, JNK and p38 MAPK in fibroblast-like synoviocytes stimulated by TNF-α. Furthermore, HSP70 also significantly inhibited nuclear translocation of nuclear factor-κB and degradation of IκBα induced by TNF-α.

**Conclusion:**

Extracellular HSP70 has an anti-inflammatory effect on RA by downregulating production of IL-6, IL-8 and MCP-1 in fibroblast-like synoviocytes, which is mediated through inhibited activation of the MAPKs and NF-κB signal pathways.

## Introduction

Rheumatoid arthritis (RA) is a chronic disease that is characterized by inflammation of the synovial membrane and proliferation of the synovial lining, resulting in progressive joint destruction [1]. Fibroblast-like synoviocytes (FLSs) play a crucial role in the joint inflammation and destructive process [2]. RA FLSs respond to several proinflammatory cytokines, including IL-1, tumour necrosis factor (TNF)-α, and exhibit characteristics of inflammatory cells that are critically involved in various aspects of rheumatoid pathophysiology [2,3]. They synthesize and secrete proinflammatory cytokines such as IL-6, and chemokines including IL-8 and monocyte chemoattractant protein (MCP)-1 [4-7], which play roles in mediating the inflammatory functions of FLSs. IL-6 is now recognized to be a master cytokine that is involved not only in the RA cytokine cascade but also in actions such as promotion of expansion and activation of T cells, differentiation of B cells, regulation of acute phase protein genes, and regulation of chemokine production [8,9]. IL-8 and MCP-1 are key mediators that are involved in the recruitment of neutrophils, monocytes and lymphocytes, and play important roles in inflammatory cell infiltration [8]. Evidence from animal models of arthritis and from RA patients has shown that blockade of these cytokines or their receptors has beneficial effects both for inflammation and joint destruction [10-12]. Therefore, inhibition of these inflammatory mediators production by FLSs might present an effective target for RA treatment.

Heat shock proteins (HSPs) are a family of highly conserved intracellular proteins that are found in all prokaryotes and eukaryotic cells. Although some HSPs are constitutively expressed, upregulation of expression is caused by exposure to a variety of cellular stressors, including heat shock, growth factors, inflammation and infection [13,14]. HSPs are typically regarded as intracellular proteins, and their primary function appears to be that of intracellular molecular chaperones, contributing to the folding of nascent proteins and denatured proteins, and thus preventing protein aggregation under stressful stimuli [15,16]. The human stress-inducible form of the 70 kDa HSP (HSP70; Genbank: NM005345) is a many-faceted molecule. In addition to serving as a intracellular chaperone, it is released from damaged cells or viable cells after stress, and has been found in the bloodstream of both healthy individuals and those suffering from autoimmune diseases and inflammatory conditions [17,18]. Recent findings indicating a role for extracellular HSP70 as a cytokine that induces secondary proinflammatory cytokine (TNF-α, IL-1 and IL-6) production may provide insight into the pathogenesis of autoimmune disease [16,19].

Elevated levels of the inducible form of HSP70 have been identified in RA synovial tissues and RA synovial fluid relative to those in patients with osteoarthritis and healthy individuals [20,21]. It is unknown whether an increase in extracellular HSP70 plays a biological role in RA, but in animal models pre-immunization with proteins of the HSP70 family, such as mycobacterial HSP70 and the glucose-regulated protein 78, protected animals from experimentally induced arthritis. In adjuvant-induced arthritis in rats it was shown that the protection conferred by mycobacterial HSP70 resulted from the induction of IL-10 producing T cells that were capable of downregulating inflammation [22-24]. However, the precise mechanism of protection by HSP70 in RA remains unclear. Findings in arthritis models raise the question of whether HSP70 could play a role in FLSs. Analyses of FLSs from human patients with RA reveal significantly elevated extracellular expression of HSP70 [25], which suggests that extracellular HSP70 may play an immunomodulatory role in FLSs. However, the interactions of HSP70 with FLSs in RA have not previously been reported. Also unknown are whether HSP70 exogenously regulates production by FLSs of RA-associated proinflammatory mediators.

In the present study we report the first analysis of the effects of extracellular human inducible HSP70 on TNF-α induced secretion by RA FLSs of the proinflammatory cytokine IL-6 and the chemokines IL-8 and MCP-1, and we elucidate the underlying mechanism. We find that human HSP70 inhibits TNF-α induced IL-6, IL-8 and MCP-1 secretion by human RA FLSs. Furthermore, we demonstrate that human HSP70 suppresses the activation of nuclear factor-κB (NF-κB) and mitogen-activated protein kinase (MAPK) signalling pathways induced by TNF-α. These findings clearly reveal an anti-inflammatory effect of human HSP70 on TNF-α-mediated inflammation and demonstrate its mechanism in RA FLSs.

## Materials and methods

### Cell culture

FLSs were isolated from RA synovial tissues obtained at joint replacement surgery, as previously described [26]. The diagnoses of RA conformed to the 1987 revised American Council of Rheumatology criteria [27]. Briefly, tissue samples were minced and treated with 1 mg/ml collagenase for 1 to 2 hours at 37°C. After washing, the cells were cultured in Dulbecco's modified Eagle's medium (Life Technologies, Rockville, MD, USA) supplemented with 10% heat-inactivated foetal calf seum (Life Technologies), 100 IU/ml penicillin, 100 μg/ml streptomycin, and 2 mmol/l L-glutamine in a humidified incubator with 5% carbon dixoide and 95% air. After overnight culture nonadherent cells were removed, and adherent cells were cultivated in Dulbecco's modified Eagle's medium plus 10% foetal calf seum. At confluence, cells were trypsinized, split at a 1:3 ratio and re-cultured in the same medium. All the experiments described here utilize FLSs between the fourth and ninth passage. That the population of FLSs was homogeneous was determined using flow cytometry (<1% CD11b, <1% phagocytic and <1% Fcγ receptor type II positive).

### Cytokine quantification by ELISA

Following stimulation of human RA FLSs by TNF-α (R&D, Minneapolis, MN, USA) in the presence or absence of recombinant human inducible HSP70 (StressGen, Victoria, British Columbia, Canada; catalog# ESP555), supernatants were harvested. IL-6, IL-8 and MCP-1 levels were measured using a sandwich ELISA, following the manufacturer's instructions (R&D). All data were normalized by cell number.

### Preparation of cellular extracts

Following treatment with TNF-α, cells were harvested, washed twice with cold phosphate-buffered saline (PBS), and nuclear and cytoplasm extracts were prepared in accordance with the method proposed by Edgar and coworkers [28]. Briefly, the cell pellet was resuspended in 200 μl cold buffer A (10 mmol/l HEPES [pH 7.9], 10 mmol/l KCl, 0.1 mmol/l EDTA, 0.1 mmol/l EGTA, 1 mmol/l DTT and 0.5 mmol/l phenylmethylsulphonyl fluoride [PMSF]). The cells were allowed to swell on ice for 15 minutes, after which 25 μl of a 10% solution of NP-40 was added and the tube was vortexed for 10 seconds. The homogenate was centrifuged for 30 seconds in a microfuge to recover the cytoplasm extract in the supernatant. The nuclear pellet was resuspended in 50 μl ice-cold buffer B (20 mmol/l HEPES [pH 7.9], 0.4 mol/l NaCl, 1 mmol/l EDTA, 1 mmol/l EGTA, 1 mmol/l DTT and 1 mmol/l PMSF) and the tube was vigorously rocked at 4°C for 15 minutes on a shaking platform. The nuclear homogenate was centrifuged for 5 minutes to recover the nuclear extract in the supernatant. The aliquots were stored at -80°C. The protein concentrations of the fractions were determined using a standard Bradford assay.

### Western blot analysis

The following antibodies were used in this study: rabbit anti-phospho-JNK (c-Jun amino-terminal kinase) polyclonal antibody (Cell Signaling, Danvers, MA, USA; 1:1000); rabbit anti-phospho-p38 polyclonal antibody (Cell Signaling; 1:1000); sheep anti-phospho-Erk1/2 (extracellular signal-regulated protein kinase-1/2) polyclonal antibody (Upstate, Lake Placid, NY, USA; 1:1000); rabbit anti-NF-κB (nuclear factor-κB) p65 polyclonal antibody (Santa Cruz, Santa Cruz, CA; 1:200); rabbit anti-JNK polyclonal antibody (Cell Signaling; 1:1000); rabbit anti-p38 polyclonal antibody (Cell Signaling; 1:1000); rabbit anti-Erk1/2 polyclonal antibody (Cell Signaling; 1:1000); rabbit anti-IκBα polyclonal antibody (Santa Cruz; 1:1000); mouse anti-GAPDH (glyceraldehyde 3-phosphate dehydrogenase) monoclonal antibody (Upstate; 1:1000); and mouse anti-PCNA (proliferating cellular nuclear antigen) monoclonal antibody (Upstate; 1:1000), and mouse anti-tublin monoclonal antibody (Upstate; 1:1000).

Aliquots of either subcellular fractions or total cell lysates were reduced and denatured by boiling in SDS sample buffer (2× = 100 mmol/l Tris-HCl [pH 6.8]; 4% weight/vol SDS; 20% glycerol; 200 mmol/l DTT; 0.1% weight/vol bromophenol blue), fractionated on 12% SDS-PAGE, and transferred to a nitrocellulose membrane (Promega, Madison, WI, USA). Membranes were blocked in blocking buffer (2% bovine serum albumin [BSA], 0.2% Tween-20 in Tris-buffered saline) at room temperature for 4 hours, and incubated for 2 hours at 25°C with the indicated primary antibody, followed by peroxidase-conjugated secondary antibody IgG (anti-rabbit or anti-mouse [Boster Biotech, Wuhan, China; 1:1000], anti-sheep [KPL, Gaithersburg, MD, USA; 1:1000]) for 1 hours at 25°C. The signals were visualized by DAB detection (Boster Biotech), following the manufacturer's instruction, and the bands of interest were scanned and counts quantitated with the Band Leader software (Shanghai, China).

### Immunocytochemical analysis

FLSs (5 × 10^5 ^cells), cultured on glass coverslips, were fixed in 4% formaldehyde for 30 minutes at room temperature before detergent extraction with 0.1% Triton X-100 for 10 minutes at 4°C. Coverslips were blocked in PBS containing 2% BSA for 1 hour at room temperature and processed for immunofluorescence with rabbit anti-NF-κB/p65 polyclonal antibody (Santa Cruz Biotechnology, Inc., Santa Cruz, CA, USA; 1:50) followed by Cy3-conjugated sheep anti-rabbit IgG (Santa Cruz; 1:100) and Hoechst 33258 (Sigma, St. Louis, MO, USA; 1 μg/ml). Between all incubation steps, cells were washed three times for 30 minutes with PBS containing 0.2% BSA. Coverslips were mounted on slides using Movio (Sigma). Fluorescence signals were analyzed by fluorescent microscopy (Nikon, Tokyo, Japan).

### Statistical analysis

Data in the figures and text were expressed as means ± standard deviation. *P *< 0.05 was deemed to represent statistical significance, and the significance of differences between groups was determined using two-tailed Student's *t*-test or Fisher's least significant difference test.

## Results

### Human HSP70 inhibits TNF-α induced IL-6, IL-8 and MCP-1 secretion in FLSs

**Figure 1:**
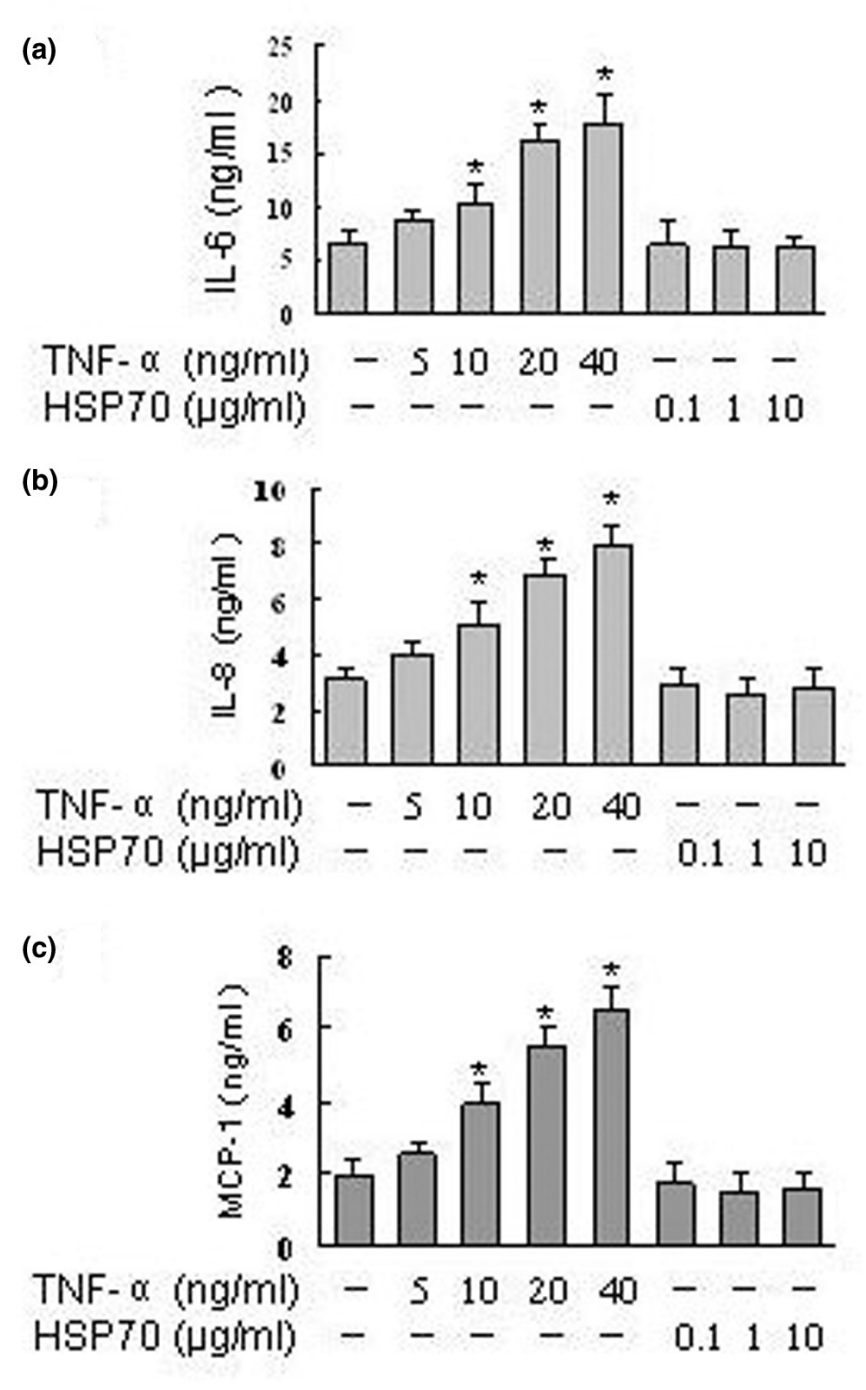
HSP70 alone has no effect on IL-6, IL-8 and MCP-1 secretion in RA FLSs. Rheumatoid arthritis fibroblast-like synoviocytes were incubated with tumour necrosis factor (TNF)-α or heat shock protein (HSP)70 at the indicated concentrations for 24 hours. The **(a) **IL-6, **(b) **IL-8 and **(c) **monocyte chemoattractant protein (MCP)-1 concentrations in the culture supernatants were determined using ELISA. Data are presented as means ± standard deviation of three independent experiments. *P < 0.05 versus unstimulated control group.

**Figure 2:**
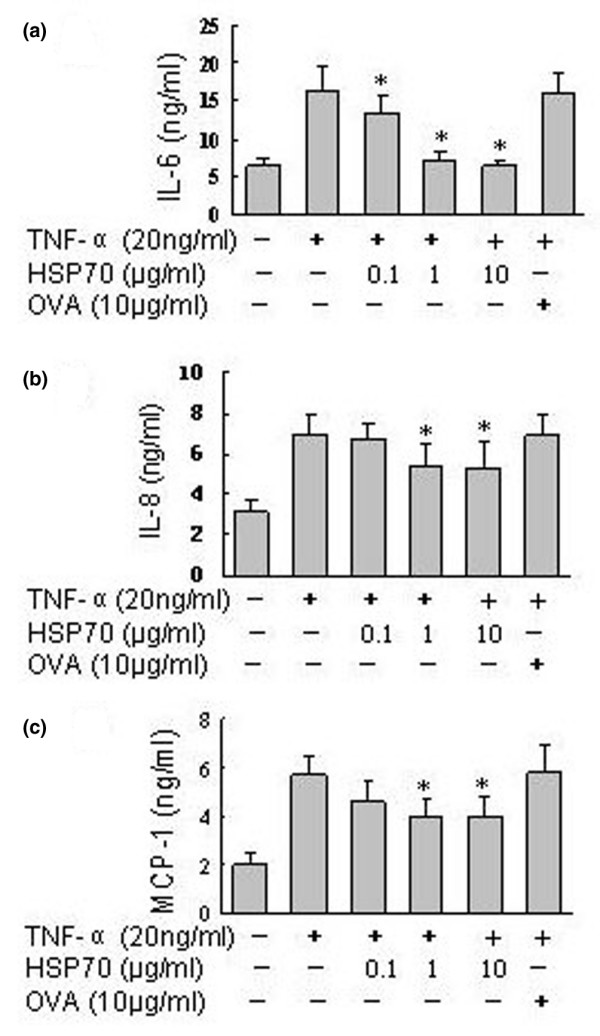
HSP70 inhibits TNF-α induced IL-6, IL-8 and MCP-1 secretion in RA FLSs. Rheumatoid arthritis (RA) fibroblast-like synoviocytes (FLSs) were incubated with the indicated concentrations of heat shock protein (HSP)70 or control protein OVA (10 μg/ml) for 1 hour, washed and then exposed to tumour necrosis factor (TNF)-α (20 ng/ml). The supernatants were harvested after 24 hours, and **(a) **IL-6, **(b) **IL-8 and **(c) **monocyte chemoattractant protein (MCP)-1 concentrations were determined using ELISA. Data are presented as means ± standard deviation of three independent experiments. *P < 0.05 versus the TNF-α stimulated group.

It has been demonstrated that IL-6, IL-8 and MCP-1 are key proinflammatory mediators, produced mainly by FLSs in the synovium, and play crucial roles in the pathophysiology of RA [29]. TNF-α is a potent activator of production of these proinflammatory mediators in FLSs [8,30]. We therefore analyzed the effects of human HSP70 on TNF-α induced secretion of IL-6, IL-8 and MCP-1 in RA FLSs. As demonstrated in Figure 1, TNF-α stimulation (5 to 40 ng/ml) for 24 hours induced a dose-dependent increase in IL-6, IL-8 and MCP-1 secretion by the RA FLSs. Peak levels of IL-6, IL-8 and MCP-1 production were noted with 20 to 40 ng/ml TNF-α. In contrast to TNF-α, human HSP70 (0.1 to 10 μg/ml) alone had no significant effects on secretion by RA FLSs of IL-6, IL-8 and MCP-1 (Figure 1). However, when the FLSs were pretreated with different concentrations of human HSP70 for 1 hour, washed and then exposed to TNF-α (20 ng/ml) for 24 hours, secretion of IL-6, IL-8 and MCP-1 was inhibited. As shown in Figure 2, levels of production of IL-6, IL-8 and MCP-1 were increased after TNF-α stimulation as compared with untreated controls, and the TNF-α induced increases in IL-6, IL-8 and MCP-1 secretion were attenuated in cells treated with HSP70. The inhibitory effects of HSP70 on IL-6, IL-8 and MCP-1 secretion were dose dependent, with prominent effects occurring at 1 to 10 μg/ml HSP70. However, treatment with the control protein ovalbumin [31] did not inhibit TNF-α induced IL-6, IL-8 and MCP-1 secretion (Figure 2). In addition, similar results were found when we conducted the same experiment without washing the FLSs between HSP70 and TNF-α stimulation (Additional file 1).

**Figure 3:**
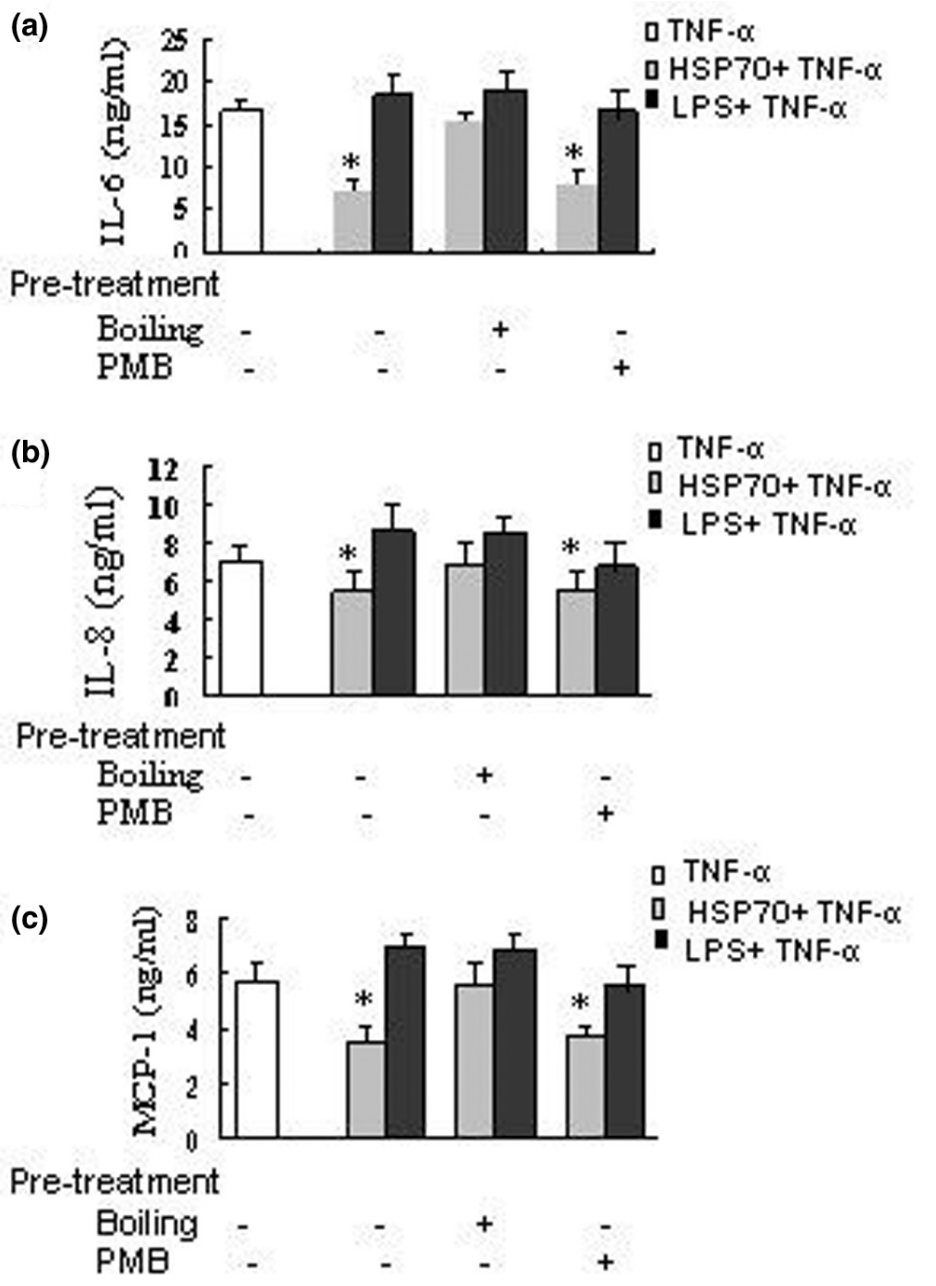
Effects of human HSP70 and lipopolysaccharide. The effects of human heat shock protein (HSP)70 on proinflammatory mediator secretion in fibroblast-like synoviocytes (FLSs) are not due to contaminating lipopolysaccharide (LPS). Rheumatoid arthritis (RA) FLSs were incubated with untreated, polymyxin B (PMB; 10 μg/ml)-treated, or boiling(100°C, 30 minutes)-treated human heat shock protein 70 (HSP70;1 μg/ml) or lipopolysaccharide (LPS; 100 ng/ml) for 1 hour, washed and then exposed to tumour necrosis factor (TNF)-α (20 ng/ml). The supernatants were harvested after 24 hours, and **(a) **IL-6, **(b) **IL-8 and **(c) **monocyte chemoattractant protein (MCP)-1 concentrations were determined using ELISA. Data are presented as means ± standard deviation of three independent experiments. *P < 0.05 versus TNF-α alone.

Recent studies have shown that contamination of HSP70 with lipopolysaccharide might be responsible for its stimulatory activation on macrophages and dendritic cells [32,33]. To test whether a contamination of our HSP70 preparation with lipopolysaccharide might have been responsible for the observed effects of HSP70 on cytokine secretion by FLSs, we applied a kinetic-turbidimetric method. We first observed that the recombinant human HSP70 used in this study contained under 0.01 EU/μg protein (1 pg/μg) bacterial endotoxin. Second, the following studies were conducted to exclude the possibility that such minute amounts of lipopolysaccharide might affect cytokine secretion by FLSs by using the lipopolysaccharide inhibitor polymyxin B and by boiling the HSP70. Figure 3 shows that the effects of human HSP70 on IL-6, IL-8 and MCP-1 secretion were completely inhibited by boiling (which denatures proteins but not lipopolysaccharide) but not by polymyxin B, whereas the effects of lipopolysaccharide (100 ng/ml) on IL-6, IL-8 and MCP-1 secretion were inhibited by polymyxin B but not by boiling. In addition, in contrast to the inhibitory effects of HSP70 on FLSs, lipopolysaccharide exhibited slightly stimulatory effects on IL-6, IL-8 and MCP-1 secretion by FLSs (Figure 3). Consequently, we conclude that the effects of HSP70 on proinflammatory mediator secretion in FLSs were not influenced by possible lipopolysaccharide contamination in the preparation of HSP70.

### Human HSP70 suppresses the phosphorylation of MAPKs induced by TNF-α in FLSs

**Figure 4:**
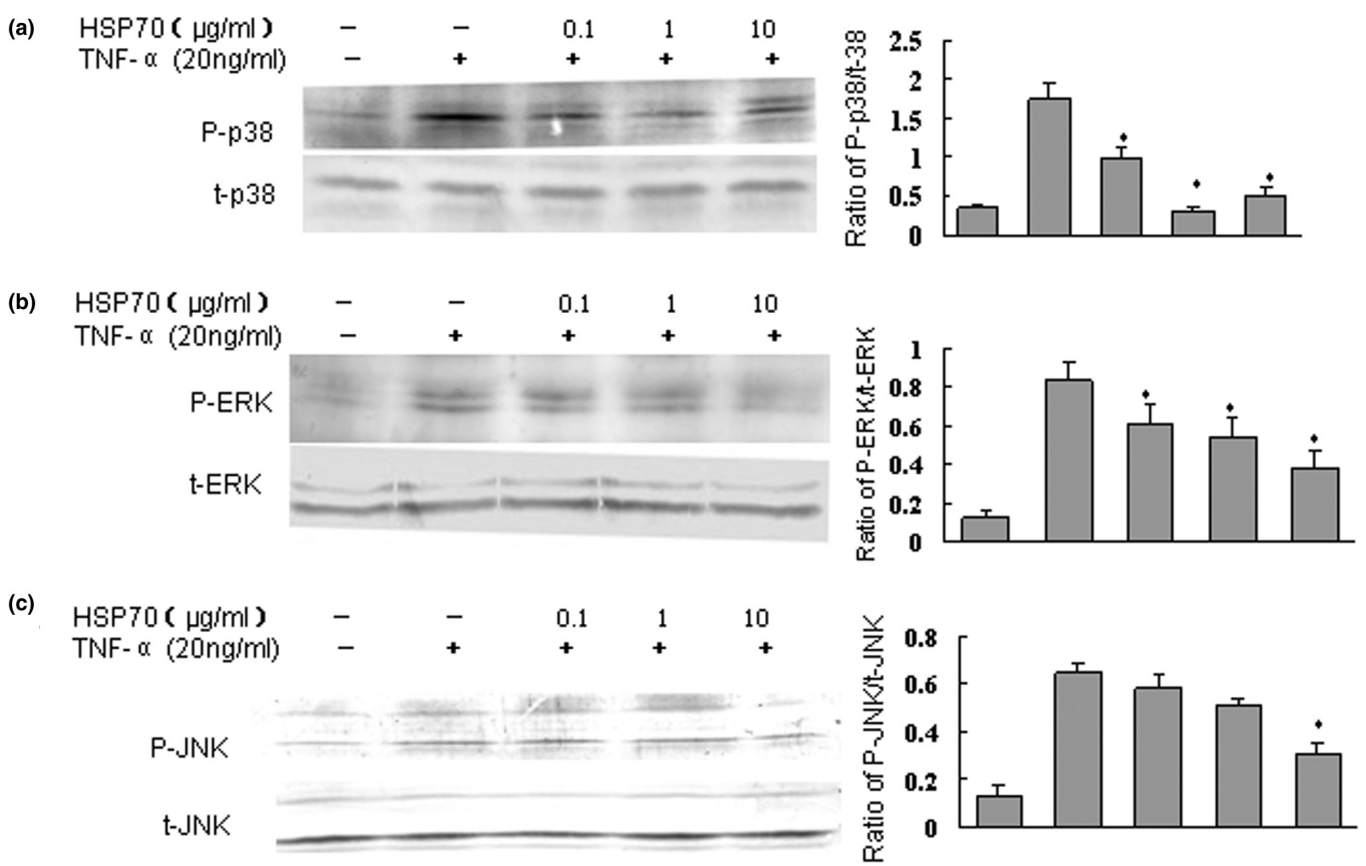
HSP70 suppresses TNF-α induced phosphorylation of MAPKs in human FLSs. Rheumatoid arthritis (RA) fibroblast-like synoviocytes (FLSs) were incubated with heat shock protein (HSP)70 at 0.1 to 10 μg/ml for 1 hour, and the FLSs were washed and exposed to tumour necrosis factor (TNF)-α (20 ng/ml) for 30 minutes. The cell lysates were immunoblotted with **(a) **anti-phospho-p38 (p-p38) and anti-total p38 (t-p38), **(b) **anti-phospho-ERK (p-ERK) and anti-total ERK (t-ERK), and **(c) **anti-phospho-JNK (p-JNK) and anti-total JNK (t-JNK). Antibodies (Abs) against t-p38, t-ERK, or t-JNK served as controls. The levels of p38, extracellular signal-regulated protein kinase (ERK), and c-Jun amino-terminal kinase (JNK) were estimated by densitometry. Shown in the left panels are representative Western blots, and in the right panels are presented the means ± standard deviation of three independent experiments. *P < 0.05 versus the TNF-α group. MAPK, mitogen-activated protein kinase.

TNF-α-induced inflammatory cytokine production by FLSs involves the activation of three MAPKs, namely p38, ERK1/2 (p44/42) and JNK (p46/54) [34]. To understand fully the mechanism by which HSP70 inhibits TNF-α induced proinflammatory mediator production by human RA FLSs, we first investigated the possible effects of HSP70 on the phosphorylation of p38, ERK and JNK. After cells were stimulated by TNF-α, phosphorylation levels of MAPKs were subsequently measured by Western blotting analysis using three different kinds of phospho-specific antibodies. The results indicate that the phosphorylation levels of all three MAPKs increased dramatically after 30 minutes of treatment with TNF-α (20 ng/ml), which is consistent with previous reports [34]. However, the phosphorylation of all three MAPK was inhibited when FLSs were pretreated with human HSP70 and then exposed to TNF-α for 30 minutes (Figure 4). The inhibitory effects occurred in a dose-independent manner; maximal inhibition was achieved with 1 to 10 μg/ml HSP70. Moreover, HSP70 inhibition of the phosphorylation of p38 MAPK was more significant as compared with its inhibition of JNK and ERK. Without TNF-α stimulation, HSP70 alone did not significantly affect the phosphorylation of the MAPKs in RA FLSs (date not shown). Thus, the inhibitory effects of human HSP70 on proinflammatory mediator secretion induced by TNF-α in FLSs could be attributed to the suppression of MAPK pathways.

### Human HSP70 inhibits nuclear translocation of NF-κB induced by TNF-α in FLSs

**Figure 5:**
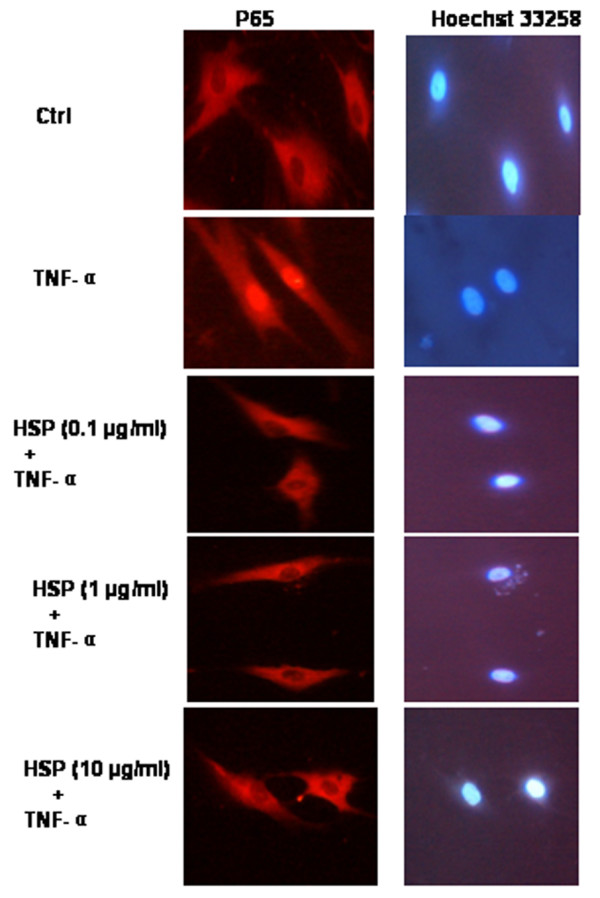
HSP70 inhibits TNF-α induced nuclear translocation of NF-κB in FLSs, as detected using immunocytochemistry. Rheumatoid arthritis (RA) fibroblast-like synoviocytes (FLSs) were incubated with heat shock protein (HSP)70 (1 μg/ml) for 1 hour, and the FLSs were washed and exposed to tumour necrosis factor (TNF)-α (20 ng/ml) for 30 minutes. The cells were fixed, permeabilized and incubated with rabbit anti-p65 antibody, followed by Cy3-conjugated anti-rabbit immunoglobulin (red). The nuclei of the corresponding cells were demonstrated by Hoechst 33258 staining. Total magnification for images was 200×.

Because activation and nuclear translocation of NF-κB is an essential step in the regulation of production of cytokines [35], we first examined whether HSP70 could inhibit the TNF-α induced nuclear translocation of NF-κB by immunofluorescence. We found that p65 subunit of NF-κB was distributed in the cytoplasmic compartment in all cells before TNF-α stimulation. Treatment with TNF-α (20 ng/ml) resulted in marked accumulation of p65 in nuclei after 30 minutes. However, nuclear translocation of p65 induced by TNF-α was significantly inhibited in cells pretreated with HSP70 (Figure 5). HSP70 alone, even at high concentrations (up to 10 μg/ml), could not induce NF-κB nuclear translocation at all (date not shown).

**Figure 6:**
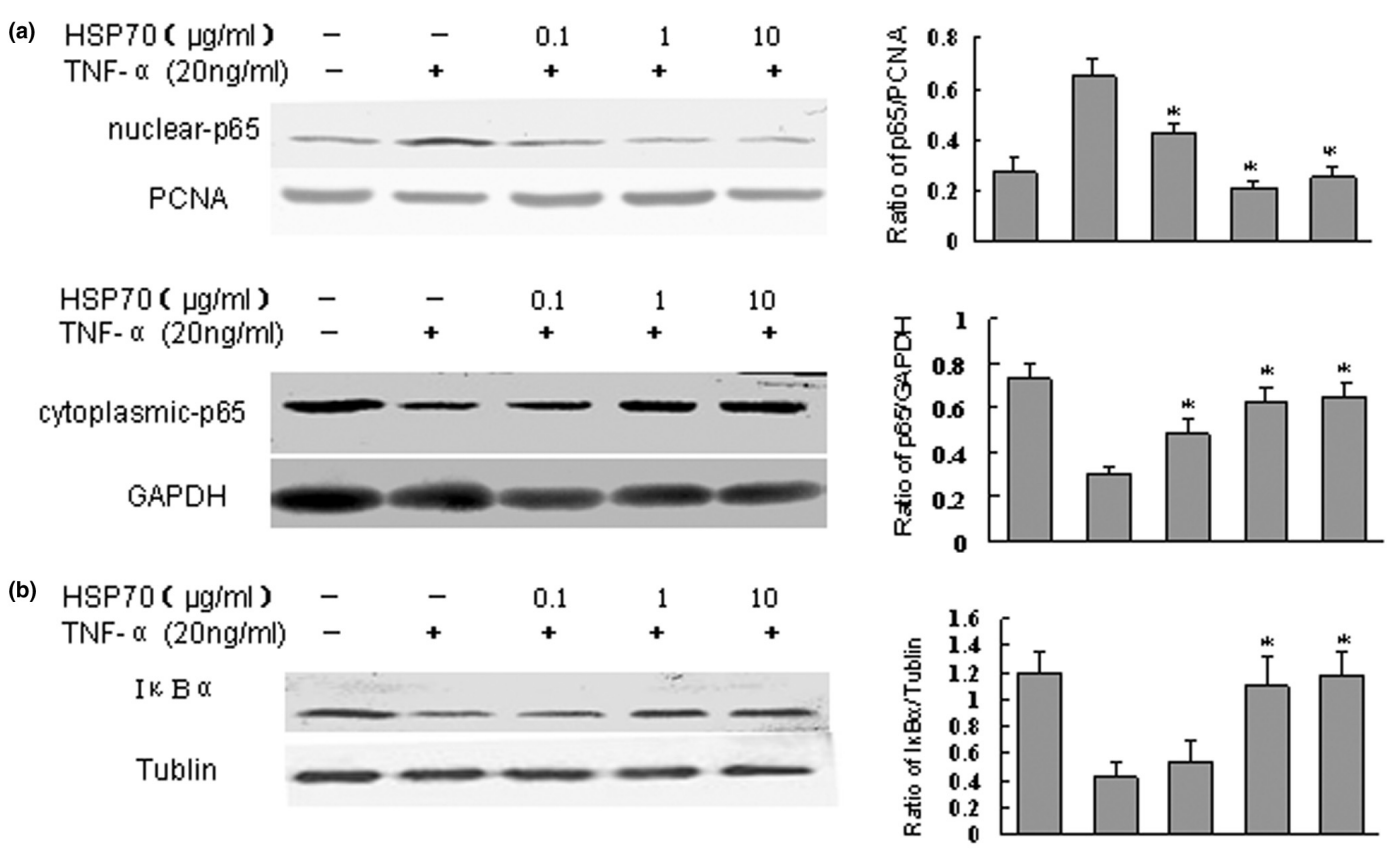
HSP70 inhibits translocation of NF-κB and degradation of IκBα. Heat shock protein (HSP)70 inhibits tumour necrosis factor (TNF)-α induced nuclear translocation of nuclear factor-κB (NF-κB) and degradation of IκBα in fibroblast-like synoviocytes (FLSs) detected by Western blot. **(a) **Human rheumatoid arthritis (RA) FLSs were incubated with HSP70 at 0.1 to 10 μg/ml for 1 hour, and the FLSs were washed and exposed to TNF-α (20 ng/ml) for 30 minutes. Nuclear or cytoplasmic lysates were immunoblotted with anti-P65, anti-PCNA (proliferating cellular nuclear antigen), or anti-GAPDH (glyceraldehyde 3-phosphate dehydrogenase). PCNA and GAPDH served as controls for nuclear and cytoplasmic proteins. The levels of p65, GAPDH and PCNA were estimated by densitometry. Shown in the left panels are representative Western blots, and shown in the right panels are the means ± standard deviation of three independent experiments. *P < 0.05 versus the TNF-α group. **(b) **Human RA FLSs were incubated with HSP70 at 0.1 to 10 μg/ml for 1 hour. Then, the FLSs were washed and exposed to TNF-α (20 ng/ml) for 20 minutes. Cytoplasmic lysates were immunoblotted with IκBα or anti-tubulin. Tubulin served as a control for cytoplasmic protein. The levels of IκBα and tubulin were estimated by densitometry. Shown in the left panel is a representative Western blot, and shown in the right panel are the means ± standard deviation of three independent experiments. *P < 0.05 versus the TNF-α group.

We further confirmed these results using a Western blotting approach by probing nuclear and cytoplasmic FLS cell extracts using monoclonal antibodies specific for the p65 subunit of NF-κB. The results showed that nuclear translocation of p65 from the cytoplasm to the nucleus occurred at 30 minutes after TNF-α stimulation (date not shown). Treatment of FLSs with HSP70 for 1 hours alone did not affect the nuclear translocation of NF-κB (date not shown). However, pretreatment of FLSs with human HSP70 for 1 hour, followed by exposure to TNF-α for 30 minutes, caused a significant inhibition of NF-κB translocation to the nucleus, and kept the p65 subunit of NF-κB in the cytoplasmic compartment (Figure 6a).

### Human HSP70 inhibits the TNF-α induced degradation of IκBα

In order to examine the roles played by IκBα in the NF-κB activation pathway by masking the nuclear localization sequence, we investigated the degradation of IkBα by Western blotting using antibodies against IκBα. To explore the mechanism by which HSP70 inhibits the nuclear translocation of NF-κB, we determined whether HSP70 could inhibit the TNF-α induced degradation of IκBα. TNF-α (20 ng/ml) markedly induced degradation of IκBα at 20 minutes, and this degradation was significantly inhibited in cells pretreated with human HSP70 (Figure 6b). It was therefore concluded that HSP70 can inhibit the degradation of IκBα and subsequent nuclear translocation of NF-κB induced by TNF-α.

## Discussion

It has been demonstrated that the HSP70 family of proteins can downregulate adjuvant arthritis [24], and it is likely that the protection resulted from induction of self-HSP70 cross-reactive T cells that are capable of downregulating inflammation [22]. However, the mechanism underlying the regulatory effect of HSP70 in RA is complex and incompletely understood. Synovial FLSs play a vital role in both chronic inflammation and joint destruction, principally through synthesis of proinflammatory cytokines and chemokines [36], which play essentially pathogenetic roles in RA. In the present study we investigated – for the first time – the effects of human HSP70 on secretion of the proinflammatory cytokine IL-6 and chemokines IL-8 and MCP-1 by human RA FLSs. Our results clearly demonstrated that human HSP70 suppressed TNF-α induced IL-6, IL-8 and MCP-1 production in a dose-dependent manner in human RA FLSs. Moreover, we observed that HSP70 suppressed the activation of the proinflammatory mediator associated NF-κB and MAPKs signalling pathways induced by TNF-α. Based on these combined observations, we conclude that extracellular human HSP70 has anti-inflammatory effects on RA, probably due to inhibitory effects of HSP70 on production of proinflammatory cytokines and chemokines in FLSs.

The roles played by HSP70 in the immune response have been a focus of many recent investigations [24,37]. HSP70 has been reported to have both proinflammatory and anti-inflammatory effects in autoimmune diseases [16]. Mammalian and bacterial HSP70 have been described to activate antigen-presenting cells directly, including macrophages and dendritic cells [17]. Such immune activation might contribute to breaking of tolerance to autoantigens, leading to the induction of autoimmune disease [16]. HSP70 can also regulate autoimmunity indirectly by activating regulatory T cells that control pathogenic T cells specific for self-antigens other than HSPs [24]. HSP70 has also been studied and identified as an immune target in RA. The HSP70 family of proteins has been implicated in the pathogenesis of experimental and human arthritis. Immunization with mycobacterial HSP70 has been found to protect rats from experimentally induced arthritis through induction of IL-10 producing T cells [22,23,38]. Intravenous or subcutaneous administration of the endoplasmic reticulum chaperone BiP (a mammalian HSP70 family member) was also found to prevent induction of and to treat ongoing collagen-induced arthritis [39]. Elevated levels of antibody to HSP70 have been reported in RA, and the level of HSP70 has been shown to be enhanced in RA synovial fluid and synovial tissue [20,21,40].

Although studies on the role of extracellular HSP70 in human RA are incomplete, a picture is emerging in which the expression of HSP70 or immune reactivity to HSP70 in RA appears to be associated with downregulation, rather than induction or propagation of inflammation. It was recently shown that mycobacterial HSP70 treatment *in vitro *induced IL-10 production in monocytes from blood and synovial tissue from arthritis patients [41]. Also, BiP stimulation of human peripheral blood mononuclear cells *in vitro *was found to trigger the production of anti-inflammatory cytokines [42]. However, to our knowledge, the effect of extracellular human HSP70 on human RA FLSs has not previously been studied.

The present study revealed that human HSP70 inhibited the IL-6, IL-8 and MCP-1 expression in RA FLSs induced by TNF-α stimulation (Figure 2), although HSP70 alone had no effect on FLSs (Figure 1). The findings suggest that self-HSP70 may have anti-inflammatory effects on RA, which might partly be due to downregulation of proinflammatory mediators by FLSs. However, HSP70 has also been shown to induce proinflammatory cytokines such as TNF-α, IL-1 and IL-6 in monocytes and macrophages [16,43]. The explanation for these differences may lie in the different types and/or activation status of cells used for the experiments. Interestingly, similar extracellular anti-inflammatory functions for other self-HSPs have also been suggested. Treatment with human HSP60 of T cells *in vitro *was found to inhibit the production of proinflammatory cytokines TNF-α and interferon-γ, and to trigger production of the anti-inflammatory cytokine IL-10, which is mediated via Toll-like receptor 2 [44]. Treatment of human monocytes with human HSP27 exaggerated IL-10 production [45]. Treatment with human HSP10 *in vitro *inhibited lipopolysaccharide-induced activation of NF-κB; reduced secretion of lipopolysaccharide-induced TNF-α, IL-6 and RANTES (regulated on activation, normal T cell activated and secreted); and enhanced IL-10 production from human peripheral blood mononuclear cells [46].

These findings suggest that, rather than being proinflammatory, self-HSP reactivity might be a physiological mechanism for regulating proinflammatory responses and inflammatory diseases. It should therefore not be surprising if self-HSP70 were found to have an anti-inflammatory effect in RA. It has been shown that inflammatory stress induces HSP70 release from viable human FLSs and normal peripheral blood mononuclear cells [21]. It is thus conceivable that, in the inflamed RA joint, inflammatory stress contributes to the expression and release of HSP70, and the extracellular HSP70 may act as a natural dimmer of inflammation, which might regulate both T cell and FLS mediated inflammation.

Thus far there exists no information about the signalling pathways that are involved in HSP70-mediated inhibitory effects on inflammation in FLSs. Signalling pathways that regulate proinflammatory mediator expression in RA FLSs include MAPKs and NF-κB. Three MAPK families have been implicated as playing a role in RA, including ERK1/2, JNK and the p38 MAPK [34]. Interestingly, all three of these MAPK families are activated in RA synovial tissue and in cultured RA FLSs, and TNF-α has the potential to signal through all of them [47]. Our study showed that treatment with HSP70 alone had no effect on activation of the three MAPKs (date not shown). However, human HSP70 markedly inhibited TNF-α stimulated p38, ERK and JNK phosphorylation (Figure 4). This inhibition was more obvious in TNF-α stimulated p38 phosphorylation. Collectively, these date suggest that HSP70 may suppress proinflammatory mediator production in RA FLSs via suppression of MAPK pathways.

Apart from the MAPKs, NF-κB is another key regulator of proinflammatory mediator expression and plays an important role in the induction of inflammatory cytokines in primordial mesenchymal cell lineages, including lymphocytes, macrophages and fibroblasts [35,48]. NF-κB is mainly composed of two subunits – p50 and p65 – and is retained in the cytosol of nonstimulated cells by a noncovalent interaction with the inhibitory molecule IκB. Upon stimulation by proinflammatory cytokines such as TNF-α and IL-1, IκB is degradated and NF-κB is released and translocated to the nucleus to regulate inflammatory gene expression [35,47]. NF-κB is also over-expressed in RA synovium [48,49], and activated in RA FLSs in response to TNF-α and IL-1 [26,47,50]. We found that the degradation of IκB and subsequent nuclear translocation of the NF-κB subunit p65 induced by TNF-α were strongly inhibited by human HSP70. Accordingly, we conclude that the attenuation by HSP70 of proinflammatory mediator production upon exposure to TNF-α was at least partially mediated by the suppression of NF-κB pathway. The mechanism by which HSP70 inhibits the TNF-α induced degradation of IκBα remains unclear. We speculate that HSP70 in medium may bind its specific surface receptor on the RA FLSs, and activate intracellular anti-inflammatory signal transduction pathways such as JAK2-STAT3-SOCS3, which can inhibit the TNF-α induced degradation of IκB as well as subsequent activation and nuclear translocation of NF-κB. Recently, Human RA FLSs were shown to express high levels of the CD91 molecule [51], which is a known internalizing receptor for HSP70. Therefore, it is also possible that HSP70 may interact with the cell surface via the CD91 receptor, leading to receptor mediated endocytosis. Once taken up, HSP70 may function in the same way as does intracellular HSP70, which exerts its chaperone functions and inhibits degradation of IκBα.

## Conclusion

In this study we demonstrate a novel role for exogenous human HSP70 in suppressing proinflammatory mediator production by human RA FLSs. The anti-inflammatory role played by human HSP70 in human RA FLSs may be accounted for by its ability to downregulate TNF-α induced activation of MAPK and NF-κB, two vital inflammatory signal pathways in FLSs of inflammation in RA. The results of this study indicate that human HSP70, which is upregulated and released in response to stress and inflammation, can function as a downregulator of FLS-induced inflammation in RA.

## Abbreviations

BSA = bovine serum albumin; ELISA = enzyme-linked immunosorbent assay; ERK = extracellular signal-regulated protein kinase; FLS = fibroblast-like synoviocyte; GAPDH = glyceraldehyde 3-phosphate dehydrogenase; HSP = heat shock protein; IL = interleukin; JNK = c-Jun amino-terminal kinase; MAPK = mitogen-activated protein kinase; MCP = monocyte chemoattractant protein; NF-κB = nuclear factor-κB; PBS = phosphate-buffered saline; PCNA = proliferating cellular nuclear antigen; PMSF = phenylmethylsulphonyl fluoride; RA = rheumatoid arthritis; TNF = tumour necrosis factor.

## Competing interests

The authors declare that they have no competing interests.

## Authors' contributions

XZ and XX conceived of the study, participated in its design and coordination, and helped to draft the manuscript. XL conceived of the study, participated in its design and performed the statistical analysis. YZ carried out the sample collection and analysis of data. BZ carried out the ELISA analysis. YS and ML conducted the Western blot analysis. KW participated in immunocytochemical analysis. DRMcM helped to revise the manuscript.

## Supplementary Material

Additional file 1 file showing that HSP70 inhibited TNF-α induced IL-6, IL-8 and MCP-1 secretion in RA FLSs. RA FLSs were incubated with the indicated concentrations of HSP70 or control protein OVA (10 μg/ml) for 1 hour, and then exposed to TNF-α (20 ng/ml). The supernatants were harvested after 24 h, and (A) IL-6, (B) IL-8 and (C) MCP-1 concentrations were determined using ELISA. Data are expressed as means ± standard deviation of three independent experiments. *P < 0.05 versus the TNF-α stimulated group. Click here for file
