# Supplementary figures and images for: Extracellular heat shock protein 70 inhibits tumour necrosis factor-α induced proinflammatory mediator production in fibroblast-like synoviocytes
Source: Arthritis Res Ther. 2008 Apr 14;10(2):R41. doi: 10.1186/ar2399 (PMC2453760; doi:10.1186/ar2399)

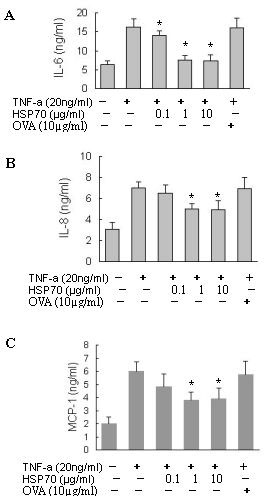

Supplement: Additional file 1 — file showing that HSP70 inhibited TNF-α induced IL-6, IL-8 and MCP-1 secretion in RA FLSs. RA FLSs were incubated with the indicated concentrations of HSP70 or control protein OVA (10 μg/ml) for 1 hour, and then exposed to TNF-α (20 ng/ml). The supernatants were harvested after 24 h, and (A) IL-6, (B) IL-8 and (C) MCP-1 concentrations were determined using ELISA. Data are expressed as means ± standard deviation of three independent experiments. *P < 0.05 versus the TNF-α stimulated group. [file ar2399-S1.jpeg]
